# Electrical Cell‐Substrate Impedance Spectroscopy Can Monitor Age‐Grouped Human Adipose Stem Cell Variability During Osteogenic Differentiation

**DOI:** 10.5966/sctm.2015-0404

**Published:** 2016-09-07

**Authors:** Rachel C. Nordberg, Jianlei Zhang, Emily H. Griffith, Matthew W. Frank, Binil Starly, Elizabeth G. Loboa

**Affiliations:** ^1^ Joint Department of Biomedical Engineering, University of North Carolina Chapel Hill and North Carolina State University, Raleigh, North Carolina, USA; ^2^ Edward P. Fitts Department of Industrial and Systems Engineering, North Carolina State University, Raleigh, North Carolina, USA; ^3^ Department of Statistics, North Carolina State University, Raleigh, North Carolina, USA; ^4^ University of Missouri College of Engineering, Columbia, Missouri, USA

**Keywords:** Electrical cell‐substrate impedance spectroscopy, Adipose stem cells, Osteogenesis, Bioimpedance

## Abstract

Human adipose stem cells (hASCs) are an attractive cell source for bone tissue engineering applications. However, a critical issue to be addressed before widespread hASC clinical translation is the dramatic variability in proliferative capacity and osteogenic potential among hASCs isolated from different donors. The goal of this study was to test our hypothesis that electrical cell‐substrate impedance spectroscopy (ECIS) could track complex bioimpedance patterns of hASCs throughout proliferation and osteogenic differentiation to better understand and predict variability among hASC populations. Superlots composed of hASCs from young (aged 24–36 years), middle‐aged (aged 48–55 years), and elderly (aged 60–81 years) donors were seeded on gold electrode arrays. Complex impedance measurements were taken throughout proliferation and osteogenic differentiation. During osteogenic differentiation, four impedance phases were identified: increase, primary stabilization, drop phase, and secondary stabilization. Matrix deposition was first observed 48–96 hours after the impedance maximum, indicating, for the first time, that ECIS can identify morphological changes that correspond to late‐stage osteogenic differentiation. The impedance maximum was observed at day 10.0 in young, day 6.1 in middle‐aged, and day 1.3 in elderly hASCs, suggesting that hASCs from younger donors require a longer time to differentiate than do hASCs from older donors, but young hASCs proliferated more and accreted more calcium long‐term. This is the first study to use ECIS to predict osteogenic potential of multiple hASC populations and to show that donor age may temporally control onset of osteogenesis. These findings could be critical for development of patient‐specific bone tissue engineering and regenerative medicine therapies. Stem Cells Translational Medicine *2017;6:502–511*

**Significance Statement**

Human adipose stem cells (hASCs) are an appealing cell source for bone tissue engineering and regenerative medicine applications because they can be obtained in high quantities via liposuction procedures and can differentiate down musculoskeletal lineages. However, a major barrier to clinical translation of hASCs is that cells from different donors have varying capacities to proliferate and differentiate. This study used electrical impedance spectroscopy to noninvasively track osteogenic differentiation of age‐grouped donors in real time, showing that age‐grouped hASCs have distinct complex impedance patterns. This method could be used to improve understanding of the biology that causes variability among hASC populations and to provide quantitative quality control standards for hASC populations in stem cell manufacturing and bone tissue engineering applications.

## Introduction

Variability among stem cells isolated from different donors is a well‐documented barrier to the clinical translation of autologous stem cell therapies 1, 2, 3, 4, 5. Human adipose stem cells (hASCs) are an attractive autologous stem cell source for tissue engineering and regenerative medicine applications because of their relative ease of isolation, multipotent differentiation capacity, and immunocompatibility 6, 7, 8, 9, 10. However, as with other stem cell sources, hASCs isolated from different donors vary dramatically in their potential to proliferate and differentiate. We have previously demonstrated that hASCs isolated from donors of different age groups have different capacities to differentiate down the osteogenic lineage 2. Other studies have documented that other demographic factors such as body mass index 11, and gender 12 also affect hASC proliferation and/or differentiation potential. We have shown that hASCs from different donors also have a variable response to mechanical stimuli 3. However, the study of donor demographic characteristics alone cannot provide a clear understanding of the underlying causes of donor variability. Furthermore, because hASCs enter widespread clinical use, it is desirable to develop a noninvasive, quantitative method to screen hASC populations for their potential use in autologous therapies and to monitor their differentiation status for quality control purposes.

Electrical cell‐substrate impedance spectroscopy (ECIS) was originally developed by Giaever and Keese 13 and has been used in a variety of applications, including monitoring cell micromotion 14, response to drugs, and assessment of barrier function 15. ECIS applies a very weak (<1 μA), noninvasive alternating current to cells seeded on a gold electrode array, allowing the cell impedance current to be monitored in real time and label‐free 14. By capturing complex impedance patterns, ECIS can evaluate dynamic aspects of cultured cells through its dielectric properties 16. In addition to providing impedance measurements, the data collected from ECIS allows additional morphology‐related parameters to be determined. Parameters that describe cell coverage, including barrier resistance (R_b_), capacitance of the cell plasma membrane (C_m_), and current flow beneath the cells (α) can be calculated 14. ECIS has previously been used to track both human bone marrow‐derived mesenchymal stem cells (hMSCs) undergoing osteogenic differentiation 17 and hASCs during osteogenic and adipogenic differentiation 18. Those studies demonstrated that hMSCs and hASCs have distinct impedance properties because they differentiate down adipogenic or osteogenic lineages. However, it is not known whether donor‐to‐donor variability can be captured via ECIS. In this study, we hypothesized that ECIS impedance properties could be used to quantify donor‐related differences in hASC populations during both proliferation and osteogenic differentiation.

In this study, we used ECIS to detect distinct bioimpedance patterns among three age‐grouped hASC superlots throughout proliferation and osteogenic differentiation. Our results demonstrate that ECIS can potentially be used to screen for osteogenic potential of hASC populations, track stages of osteogenic differentiation for quality control purposes, and better elucidate the underlying biological causes of hASC variability among donors.

## Materials and Methods

### Superlot Generation

hASCs were isolated from liposuction aspirates of female patients undergoing voluntary liposuction procedures. Complete growth medium (CGM) (minimal essential medium, α modified [Thermo Fisher Scientific Life Sciences, Waltham, MA, http://www.thermofisher.com]) supplemented with 10% fetal bovine serum (FBS) (Gemini Bio Products, West Sacramento, CA, Gemini Bio‐Products, West Sacramento), 2 mM l‐glutamine (Corning Inc., Corning, NY, http://www.corning.com), and 100 U/ml penicillin and 100 mg/ml streptomycin (penicillin‐streptomycin solution, Corning Inc.) was used to expand hASCs. Age‐grouped hASC superlots were created as described previously 2. Briefly, hASCs were pooled in equal proportions from age‐clustered donors per superlot. Age groupings consisted of young (aged 24–36 years; five donors), middle‐aged (aged 48–55 years; four donors), and elderly (aged 60–81 years; five donors) patients. The superlots were previously verified as a representative average of the individual hASC behavior by quantifying metabolic activity, calcium accretion during osteogenic differentiation, and lipid production during adipogenic differentiation 2.

### Impedance Sensing

Through use of an ECIS Zθ instrument (Applied BioPhysics, Troy, NY, http://www.biophysics.com), hASCs were seeded into eight‐well plates lined with a 40‐working electrode array to measure in real time the complex impedance values of proliferating and differentiating hASCs. Measurements were taken every 5 minutes at frequencies ranging from 10 Hz to 100 KHz. To prepare plates, each well was coated with 1 mg/ml collagen type I (Advanced BioMatrix, Inc., San Diego, CA, https://www.advancedbiomatrix.com) by covering the bottom of the plate and allowing it to sit at room temperature for 30 minutes before aspirating collagen solution off and treating with l‐cysteine (Applied BioPhysics, Inc.) for 30 minutes in the same manner, as per the recommendation of Applied BioPhysics. Cells were seeded at a density of 3,000 cells per 0.8 cm^2^ well in 400 µl of CGM. Medium was replaced every 48 hours until hASCs reached 80% confluence (determined visually by optical microscopy), at which point cell culture medium was replaced with 400 µl of CGM or osteogenic differentiation medium (ODM) (minimal essential medium, α modified (Thermo Fisher) supplemented with 10% FBS (Gemini Bio Products), 2 mM l‐glutamine (Corning Inc.), 100 U/ml penicillin and 100 mg/ml streptomycin (penicillin‐streptomycin solution, Corning Inc.), 50 µM ascorbic acid (Sigma‐Aldrich, St. Louis, MO, http://www.sigmaaldrich.com), 0.1 µM dexamethasone (Sigma‐Aldrich), and 10 mM β‐glycerolphosphate (Sigma‐Aldrich). The experiment was concluded once hASCs had undergone the impedance drop phase.

### Differentiation and Viability Characterization

Metabolic activity of the hASC populations were assessed via Alamar blue (Bio‐Rad Technologies, Raleigh, NC, https://www.bio-rad-antibodies.com) every 96 hours. DNA was quantified via Hoescht 33258 assay (Thermo Fisher). Osteogenic differentiation was assessed via calcium accretion at 14 and 21 days of differentiation using both a quantitative Calcium LiquiColor assay (Stanbio Laboratory, Boerne, TX, http://www.stanbio.com/) and alizarin red (Pacific Star Corp., Houston, TX, http://www.pfstar.com) staining as we have described previously 2, 19, 20, 21. To rule out the possibility that lipid accumulation influenced our impedance data, Oil Red O (Thermo Fisher) staining was carried out after 21 days of differentiation.

### Time‐of‐Flight Secondary Ion Mass Spectrometry Analysis

To determine the composition of observed accreted matrix, time‐of‐flight secondary ion mass spectrometry (ToF‐SIMS) was used to analyze matrix after 21 days of culture in ODM. ToF‐SIMS is a highly sensitive surface analytical technique for acquisition of elemental and molecular information from the surface of a material with high spatial and mass resolution. ToF‐SIMS analyses were conducted by using a TOF.SIMS 5 (ION TOF, Inc., Chestnut Ridge, NY, https://www.iontofusa.com/) instrument equipped with a Bi_n_^m^+ (*n* = 1 − 5, m = 1, 2) liquid metal ion gun, Cs^+^ sputtering gun, and electron flood gun for charge compensation. Both the Bi and Cs ion columns are oriented at 45° with respect to the sample surface normal. The analysis chamber pressure is maintained below 5.0 × 10^−9^ mbar to avoid contamination of the surfaces to be analyzed. For high‐lateral‐resolution mass spectral images acquired in this study, samples were sputtered for 20 cycles with Bi_3_^+^ direct current beam at 10 nA, and a burst alignment setting of 25 keV Bi_3_^+^ ion beam was used to raster a 160 μm‐by‐160 μm area. The negative secondary ion mass spectra obtained were calibrated by using cyanide ion (CN)^−^, PO_2_^−^, and PO_3_^−^. The positive secondary ion mass spectra were calibrated by using Mg^+^ and Ca^+^.

### ECIS Model

This study used the model developed by Giaever and Keese 13. When cells are confluent on the electrodes, the ECIS model decomposes the time‐course impedance data into three frequency independent parameters: (a) R_b_ (Ω.cm^2^), intercellular resistance established by tight cell‐cell junctions; (b) C_m_ (µF/cm^2^), the average cell membrane capacitance, attributed to the charge collection by the phospholipid layers of the cellular membrane; (c) α^2^ (Ω.cm^2^), indicating cell‐substrate interaction. R_b_ and α^2^ varied between age‐grouped superlots and were included within this report.

### Entropy Calculation

We used the Shannon entropy equation as a measure of signal unpredictability. The resistance portion of the impedance signal was first detrended and the signal divided into 48‐hour blocks. Shannon entropy for the resistance signal was calculated by using the following formula:

(1) $$H\left(R\right)=-\sum_{i=1}^{n}P\left(R_{i}\right)log_{2}P(R_{i})$$

where *H* indicates entropy; *i*, loop counter variable; *P*, probability of a given symbol (in this case, the probability of *R*); and *R*, resistance.

### Statistical Analysis

Turning point analysis was carried out by using PROC GLM in SAS software, version 9.4 (SAS Institute, Inc., Cary, NC, http://www.sas.com). Change points were modeled as a function of age category by using an analysis of variance model. Mean comparisons were made by using least‐squares means and post hoc testing. The calcium accretion data were summarized at day 14 and day 21. Both sets of data were analyzed by using a linear model in SAS software, version 9.4, allowing for the effects of age and trial. The Alamar blue data were analyzed by using a repeated‐measures model on the natural‐log transformed percentage reductions. Effects of day, age, trial, and a day‐by‐age interaction were included in the model. Post hoc pairwise comparisons were made by using Tukey adjustment to control the type I error rate. A repeated‐measures model was also fit to the entropy data, allowing for effects of cell culture type (ODM vs. CGM), age group, and day. In addition, paired *t* tests were calculated for the individual age groups to find the first day with a significant drop from baseline (day 0). The Bonferroni correction was used to control the type I error rate across all paired *t* tests. The significance level was set at 0.05.

## Results

### Age‐Grouped Superlots Exhibited Distinct Impedance Phases and Time‐Course Patterns During Proliferation and Osteogenic Differentiation

To determine whether ECIS could be used to rapidly detect donor‐to‐donor variability during proliferation and osteogenic differentiation, dielectric properties of hASCs were quantified in real time by seeding age‐grouped superlots onto multielectrode arrays and tracking complex impedance patterns at frequencies ranging from 10 Hz to 100 kHz throughout hASC proliferation and osteogenic differentiation. The greatest differential among hASC lines was observed at the 40‐kHz frequency. This frequency was therefore used for analysis purposes.

**Figure 1:**
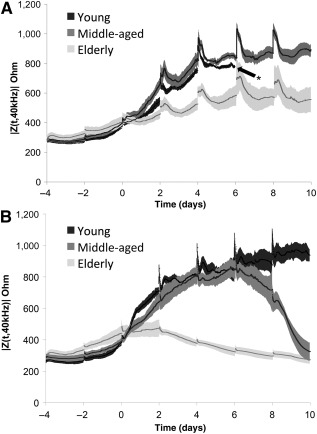
Real‐time impedance (mean ± SD) curves of human adipose stem cells (hASCs) during culture in complete growth medium (CGM) **(A)** or culture in osteogenic differentiation medium **(B)**. Osteogenic induction occurred at day 0 when applicable. ∗, Delamination of young hASC superlot cultured in CGM at day 6 media change owing to cell overgrowth.

**Figure 2:**
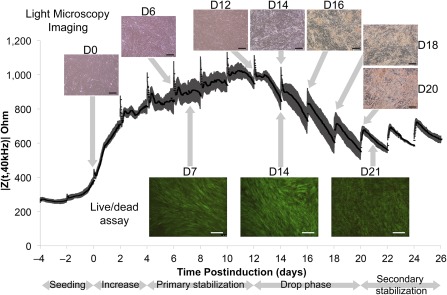
An extended impedance curve (mean ± SD) of the young superlot (human adipose stem cells [hASCs] from five female donors aged 24–36 years). The hASCs were cultured in complete growth medium until induction (day 0), at which point culture medium was changed to osteogenic differentiation medium. Matrix deposition, as identified by light microscopy, was first observed during drop phase. Full light microscopy time courses can be found in supplemental online Figures 1–3. As shown through live/dead imaging, cell viability was maintained throughout the impedance drop. Scale bars = 200 μm. Abbreviation: D, day.

**Figure 3:**
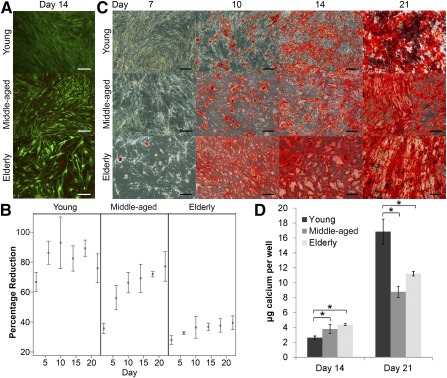
The viability and calcium accretion of superlots cultured in osteogenic differentiation medium (ODM) was assessed in parallel to electrical cell‐substrate impedance spectroscopy experiments. **(A):** Live/dead imaging of age‐grouped human adipose stem cell (hASC) superlots after 2 weeks of culture in ODM (scale bars = 200 μm). **(B):** Alamar blue profiles of superlots indicated that metabolic activity of all superlots increased or was maintained throughout culture in ODM. Greater percentage reduction correlates to higher metabolic activity. **(C):** Alazirin red staining for calcium deposition of hASCs after culture in ODM showed dense calcium deposition from all superlots (scale bars = 200 μm) but with timing of calcium accretion varying among age groups. **(D):** The calcium deposition per well (mean ± SD) was quantified via Calcium LiquiColor at 2‐ and 3‐week time points and showed that the young superlot accreted significantly less calcium at 14 days but significantly more calcium at 21 days than the middle‐aged and elderly superlots (∗, *p* < .05). Abbreviation: CN^−^, cyanide ion.

We obtained ECIS curves of hASC isolated from all three superlots during expansion in CGM (Fig. 1A). Spikes in impedance were observed at each medium change every 48 hours. Because of the small surface area of the ECIS wells, the hASCs are especially susceptible to delamination due to overcrowding. The young donor superlot proliferated, reached confluence and overgrowth, and delaminated at day 6 when cultured in CGM, characterized by the detachment and rolling up of the cell monolayer. Our results are consistent with the ECIS results of Bagnaninchi and Drummond, who observed delamination of hASC maintained in growth media approximately 6.7 days after seeding 18. The middle‐aged superlot, elderly superlot, and all superlots grown in ODM did not delaminate during the experimental period. In addition, impedance curves were generated that monitored hASC differentiation in ODM (Fig. 1B). Elderly donor cells exhibited overall lower impedance in both growth medium and differentiation medium. Because an impedance drop was observed in elderly and middle‐aged donors within the short‐term 10‐day differentiation study, the young superlot study period was extended until equilibrium at 26 days (Fig. 2A). Distinct phases in differentiation could be observed throughout osteogenesis of all age‐grouped superlots: increase, primary stabilization, drop phase, and secondary stabilization. Cells remained viable throughout drop phase. hASCs from elderly donors had a significantly shortened confluent phase, resulting in an earlier drop phase when compared with hASC isolated from younger donors. Turning point analysis showed that the impedance reached a maximum value before drop phase at day 10.0 in the young superlot, day 6.1 in the middle‐aged superlot, and day 1.3 in the elderly superlot (Fig. 2B). The age‐grouped time‐to‐maximum values were highly significant (*p* < .0001). Matrix deposition was first observed via light microscopy 48–96 hours after impedance maximum in the hASCs cultured in osteogenic differentiation medium (i.e., at day 14 in the young superlot, day 8 in the middle‐aged superlot, and day 4 in the elderly superlot) (supplemental online Figs. 1–3). ToF‐SIMS analysis revealed that the observed matrix consisted of calcium, phosphates, and organic compounds (Fig. 3).

### hASCs Maintained Viability but Display Changes in Mineralization in Correlation With Osteogenic Impedance Phases

**Figure 4:**
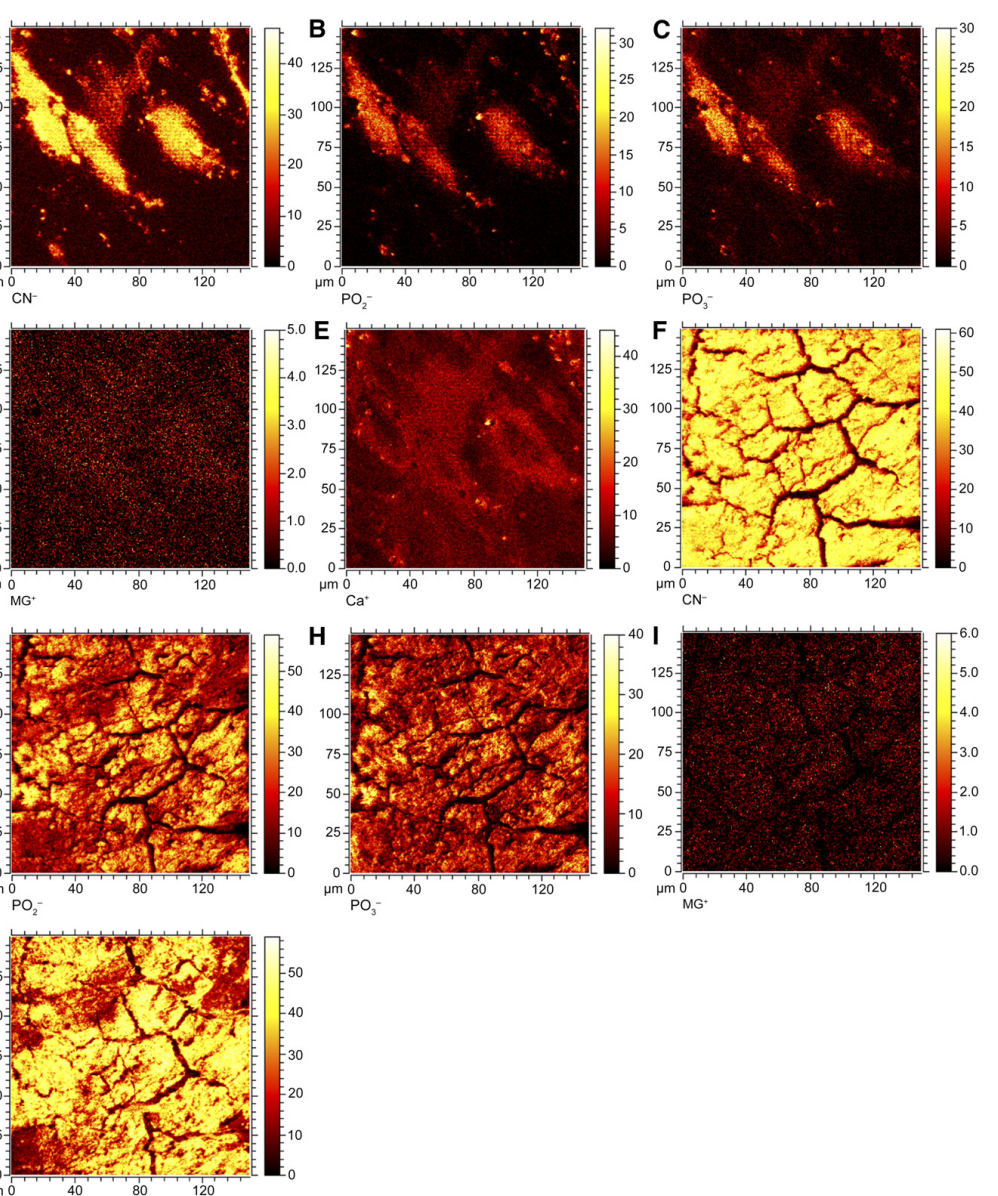
Time‐of‐flight secondary ion mass spectrometry images of deposited matrix composition after 21 days of culture in complete growth medium (CGM) **(A–E)** or osteogenic differentiation medium (ODM) **(F–J)**. The elderly superlot is represented in the images shown. Brighter intensity corresponds to greater ion detection. The cellular structures are visible on control samples cultured in CGM, and distinct matrix structure is observed in samples cultured in ODM. Images reveal that CN^−^ ions **(A, F)**, PO_2_^−^ ions **(B, G)**, and PO_3_^−^ ions **(C, H)** were observable within cellular structures in CGM, but ions cover the entire matrix surface after culture in ODM. The positive Mg^+^ ions **(D, I)** did not change significantly between control and experimental samples, but Ca^+^ ions **(E, J)** were greatly increased after culture in ODM. The field of view of each image is 160 μm × 160 μm. Abbreviation: CN, cyanide ion

To understand the underlying cause of each impedance phase, parallel studies were carried out to track the differentiation and proliferation status of the hASCs. After 2 weeks of culture in osteogenic differentiation medium, hASCs were alive in all superlots, indicating that the impedance drop had not been the result of cell death (Fig. 4A). However, a notable difference in cell density could be observed. Young hASCs had formed a dense layer of cell coverage, whereas elderly hASCs had not grown as dense and the cells appeared larger. Proliferation of hASCs in all conditions increased or was maintained throughout culture in both CGM and ODM (Fig. 3B). The interactions between metabolic activity and day and age were both highly significant (*p* < .0001). Alamar blue activity data were in accordance with DNA quantification data (supplemental online Fig. 4). Calcium accretion was tracked throughout osteogenic differentiation. hASCs from older donors appeared to exhibit slightly higher calcium accretion at 14 days, but no difference was apparent at the 21‐day time point (Fig. 4C). When quantified via Calcium LiquiColor assays, the effect of age on calcium accretion was statistically significant at both day 14 (*p* = .010) and day 21 (*p* = .002). The young hASC superlot had significantly lower calcium accretion at the 14‐day time point than both elderly (*p* = .009) and middle‐aged (*p* = .035), but at day 21 the young superlot had accreted significantly more calcium than both elderly (*p* = .005) and middle‐aged (*p* = .001) superlots (Fig. 4D). No significant difference was found between elderly and middle‐aged hASCs at day 14 (*p* = .234) or day 21 (*p* = .089). After 21 days of osteogenic differentiation, low levels of lipid deposits (<1% of cells) were observed in the young and middle‐aged superlots but no significant lipid accumulation was observed in elderly superlot (supplemental online Fig. 5).

### Distinct Membrane Resistance and Cell‐To‐Substrate Adherence Factor Were Observed Among Age‐Grouped Superlots

**Figure 5:**
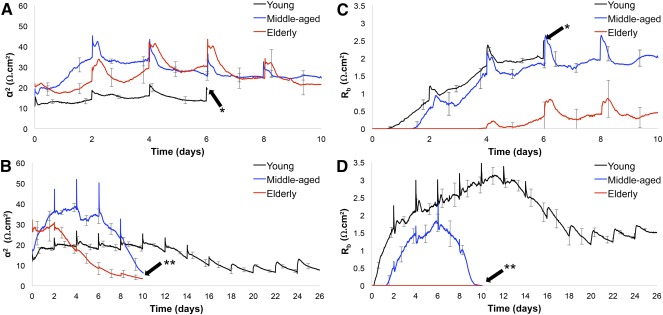
A comparison of α^2^ and R_b_ coefficients of age‐grouped superlots tracked in real time. **(A):** The α^2^ parameter of superlots cultured in complete growth medium (CGM) were lower in the young superlot than the middle‐aged or elderly superlot, indicating greater cell spreading in middle‐aged and elderly superlots. **(B):** In osteogenic differentiation medium (ODM), the α^2^ parameter was higher before differentiation, suggesting that cell spreading decreases in late‐stage osteogenic differentiation. α^2^ was highly reduced in the young and middle‐aged superlots as culture time in ODM increased, but α^2^ in the young superlot decreased only slightly as the human adipose stem cells (hASCs) were cultured in ODM. **(C):** R_b_ of the elderly superlot was greatly diminished when compared with the young and middle‐aged superlots cultured in CGM, which indicates fewer cell‐to‐cell junctions in elderly hASCs. **(D):** In ODM, elderly superlots did not establish an R_b_ coefficient during the study, indicating minimal cell‐to‐cell junctions in elderly hASCs. In the young and middle‐aged superlots, R_b_ decreased after long‐term culture in ODM, at approximately day 6 in the middle‐aged and day 11 in the young superlot. This suggests that the number and composition of intercellular junctions are altered in late‐stage osteogenic differentiation. ∗, Delamination of the young superlot in CGM. ∗∗, Point at which middle‐aged and elderly superlots were ended; at this point, they had undergone differentiation and the impedance drop phase.

The ECIS model developed by Giaever and Keese was applied to calculate the membrane resistance (R_b_) and cell‐to‐substrate adherence factor (α^2^) when the cells had completely covered the electrodes (Fig. 5). These parameters were retrieved for all superlot cell groups, and time‐course changes to Z(t,f) were observed during early induction and osteogenesis. R_b_ for the younger and middle‐aged groups continued to increase as the intercellular junctions tightened after induction. This was not seen in the elderly group of cells because a meaningful R_b_ was not detected. For the young superlots, peak R_b_ of 3.1 Ω.cm^2^ was detected on day 11 before dropping to 1.5 Ω.cm^2^ by day 26. During this period of R_b_ increase, α^2^ initially rose for 24 hours after induction, reached a peak of 21.6 Ω.cm^2^, and then remained steady until day 11. It then steadily decreased to a value of 7.5 Ω.cm^2^ by day 26. For the hASCs isolated from middle‐aged donors, a peak R_b_ of 1.8 Ω.cm^2^ was reached on day 6. Much higher α^2^ was detected for the middle‐aged hASCs when compared with younger cells. α^2^ rose for 48 hours after induction to a peak of 36.4 Ω.cm^2^ and dropped to 6.5 Ω.cm^2^ by day 10. Interestingly, R_b_ increased within 4 hours of induction for the younger group of cells, whereas it took 36 hours for R_b_ to start increasing in hASC from middle‐aged donors.

The intercellular junction resistance for hASCs in both CGM and ODM increased at roughly the same time point irrespective of culture medium type. This indicates that induction by ODM did not affect junction formation. The peak strength of the intercellular junction was higher for the younger group of cells relative to the middle‐age group. In contrast, the amount of adherence of the middle‐aged hASC superlot during the initial phases of induction was higher. Taken together, distinct dielectric properties were observed among the superlot groups, specifically with respect to differences in cell adhesion, strength of the intercellular junctions, and time at which connections began to strengthen and decline during long‐term monitoring.

### Micromotion of the hASCs Decreased During Osteogenic Differentiation

**Figure 6:**
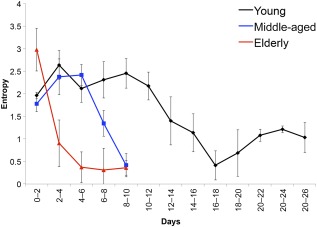
Entropy of the impedance signal was quantified throughout culture in osteogenic differentiation medium. Entropy decreased as superlots differentiated and correlated to the impedance drop phase. Entropy decreased significantly from day 0 at day 2 in the elderly (*p* < .0001), day 8 in the middle‐aged (*p* < .0001), and day 16 in the young (*p* = .0003) superlots. This indicates that micromotion of human adipose stem cells is decreased during late‐stage osteogenic differentiation.

Cellular motility of hASC superlots was evaluated by analyzing the resistance signal obtained from the real component of the impedance signal. Noise in the signal is attributed to vertical fluctuations of the cellular membrane on the electrode and is often quantified by calculating an index obtained from the power spectral density of the signal. Because of the differences in time scales during monitoring of the superlot groups, entropy in the signal data were calculated. A larger entropy measure indicates that the noise in the data are significant, and this finding is attributed to cell motility and micromotion. When quantified, the effect of “days after osteogenic induction” on the micromotion index was statistically significant (*p* < .0001). Human ASC micromotion decreased in all superlot types at time points that correlated to the impedance drop phase (Fig. 6). The decrease occurred at different time points for each of the hASC types. The elderly hASC noise signal dropped progressively until the end of the experiment, beginning immediately after osteogenic induction. By day 2, the micromotion index had decreased significantly from day 0 (*p* < .0001). For the middle‐aged group, the micromotion first significantly increased at day 2 (*p* = .002) and day 4 (*p* = .003), before a drop in micromotion was observed. The drop was first apparent at day 6, although it was not significantly lower than day 0 until day 8 (*p* < .0001). Micromotion of the young hASC superlot also increased at day 2 (*p* = .014) and began dropping at day 12 (*p* = .047). However, after accounting for multiple testing, significance from day 0 was not achieved until day 16 (*p* = .0003). Supplemental online Figure 6 shows that during proliferation in CGM, entropy measured across all superlots was high and there was no significant difference among age‐grouped superlots, but the medium (CGM vs. ODM) had a statistically significant interaction with micromotion index (*p* = .002).

## Discussion

This is the first study to monitor age‐grouped hASC proliferation and osteogenic differentiation using ECIS. We hypothesized that ECIS could be used to elucidate donor age‐related differences in dielectric properties of hASC proliferation and osteogenic differentiation in real time, thus providing a means to better understand and predict variability among hASC cell lines. We have demonstrated, for the first time, that age‐grouped superlots of hASC exhibit distinct complex impedance patterns.

We further determined a general impedance pattern observed in the differentiation of all hASC superlots that consisted of four major phases: initial increase, primary stabilization, drop phase, and secondary stabilization. Parallel tracking of hASC through traditional methods offered some explanation for these patterns. As visualized by light microscopy, hASCs proliferated after initial seeding until confluence, which correlated to increasing impedance values. In addition, this phase corresponded to increasing proliferation of the hASCs as measured via Alamar blue. Once cells reached confluence, impedance stabilized for a period of time. Eventually, an impedance drop was observed, which appeared to precede matrix deposition and mineralization by 48 to 96 hours. This suggests that cells undergo morphologic changes during late‐stage differentiation and that these changes can be detected via ECIS. This drop was not caused by cell death because cells maintained viability throughout the mineralization phase, as confirmed through live/dead assays. However, throughout osteogenic differentiation, morphologic changes resulted in less dense cell coverage and correspondingly fewer cell‐to‐cell junctions, as indicated by the dropping Rb values. Because the impedance drop preceded matrix deposition, the matrix protein interference with impedance readings alone cannot explain the impedance drop. As observed in the young hASC superlot, impedance eventually reached a secondary stabilization phase, at which time the cell impedance data stabilized while cells continued to accrete matrix. To our knowledge, this is the first time a study has shown that ECIS can be used to track specific stages of hASC osteogenic differentiation.

In this study, our goal was to use ECIS to better understand variability among hASC populations. We found the greatest separation in impedance values among age groupings at 40 kHz, at which the frequency is affected mostly by cell coverage. Our observations also hold true across a wide range of frequencies. Very similar impedance trends were witnessed in both higher and lower frequencies, and normalized curves have been included in supplemental online Figure 7. We postulate that the similarities between high and low frequencies are because cell coverage is correlated to the degree of intercellular junctions. Future studies are needed to further elaborate upon optimization of frequency range to detect specific morphological changes in differentiating stem cells.

Although matrix deposition and mineralization occur simultaneously as the hASCs undergo osteogenesis, the two processes are distinct and can have temporal variation. During endochondral ossification in vivo, collagen mineralizes to form bone 22. Here, we detected mineralization slightly before matrix deposition is observed, but more robust mineralization is detected after matrix deposition has begun. Our ToF‐SIMS analysis suggested that the matrix is a combination of both organic molecules and ionic compounds. The observable matrix in this study was likely composed of large organic compounds, such as collagen, because calcium ions would not be visible to the eye until large‐scale crystallization occurs.

Different characteristics of the general four‐phase curve were observed for each age group. Most notably, the impedance drop was observed in osteogenic differentiation of all hASC superlots but exhibited great temporal variation. Turning point analyses determined that the time‐to‐maximum impedance was at day 10.0 in young, day 6.1 in middle‐aged, and day 1.3 in elderly hASCs. This could indicate that hASC isolated from elderly donors exhibited reduced stemness when compared with the young hASCs and are therefore induced down the osteogenic lineage more rapidly. Conversely, hASCs isolated from younger patients require longer exposure to osteogenic factors before they begin to deposit matrix and mineralize. These influences of donor age on hASCs during osteogenic differentiation were clearly recognized in real time with the ECIS approach. Such influences are not facilely identifiable by more traditional characterization techniques because data are taken only at specific points in time rather than being tracked in real time. For example, although the middle‐aged and elderly superlots showed significant differences in our turning point analysis, calcium accretion was not statistically different at both the 14‐day and 21‐day time points. Total calcium accretion from a given cell population is the result of multiple factors, such as length of time cells have been accreting calcium, the amount of calcium accreted on a per‐cell basis, and whether the cells are accreting calcium at a continuous or variable rate during the time frame of interest. Quantifying total calcium accretion at a discrete point in time does not fully elucidate the dynamic nature of differentiating hASCs, demonstrating that a real‐time method to track hASC differentiation, such as ECIS, may provide greater insight into an hASC population's ability to differentiate.

Temporal timing of the onset of osteogenic differentiation could partially explain the observed variability between hASCs isolated from different donors. Although after 2 weeks the young superlot had accreted less calcium than the middle‐aged and elderly superlots, after 3 weeks of osteogenic induction the youngest superlot had accreted significantly more calcium per well than the middle‐aged or elderly hASC superlot. Again, this suggests a delayed onset of osteogenic differentiation and mineralization in the younger hASC population, allowing more time for the cells to proliferate. A previous study used ECIS to quantify impedance throughout osteogenic differentiation of hASCs, but no impedance drop was reported 18. This could potentially be explained by temporal variation in the onset of the impedance drop. The impedance drop phase of the hASCs isolated from younger donors was identified between days 12 and 20 after induction, whereas the previous study was carried out only to day 14 after induction. The hASCs used in that previous study may not have been exposed to osteogenic factors long enough to reach the impedance drop phase of osteogenic differentiation. In addition, low levels of lipid accumulation detected via Oil Red O could have had a slight effect on impedance data. Our data showing that the young and middle‐aged superlots exhibited greater lipid production than the elderly superlot are in accordance with our previous research 2.

After osteogenic induction, immediate differences in R_b_ coefficients were observed between hASCs of different age‐grouped superlots, suggesting that cell‐to‐cell junctions may help mediate hASC differentiation. In the young superlot, stronger junctions were evident in the hASCs induced down the osteogenic lineage. These findings indicate that cell‐to‐cell junctions may play an early role in osteogenic differentiation. Adheren proteins have been previously demonstrated to correlate to differentiation stage 23. In addition, dexamethasone‐induced osteogenic differentiation has been demonstrated to downregulate expression of *N*‐cadherin and Cad11 and upregulate *R*‐cadherin 24; this corresponds to the R_b_ drop that we observed during late‐stage osteogenic differentiation. Because cell‐to‐cell junctions are a major contributor to the impedance of a cell monolayer, we would expect that as adherin expression changes throughout osteogenic differentiation impedance would also change accordingly. Staining of Cnx43 at the time of induction (supplemental online Fig. 8) showed punctate staining indicating Cnx 43 in both the young and middle‐aged superlots. However, the elderly superlot did not exhibit this staining pattern, suggesting fewer cellular junctions. Further studies are required to determine which junction proteins control ECIS impedance throughout the duration of osteogenic differentiation. In addition, future studies should examine the impedance properties of bone morphogenetic protein‐2 (BMP‐2)‐induced osteogenesis, which may yield different impedance patterns because BMP‐2‐induced osteogenesis upregulates *N*‐cadherin and Cad11 expression but downregulates *R*‐cadherin 25. The effects of cell‐to‐cell junction impedance of differentiating hASCs that we found are consistent with previous studies that demonstrated ASCs induced down the adipogenic lineage had significantly lowered impedance values than ASCs induced down the osteogenic lineage 18. Before the impedance drop phase of the middle‐age and elderly cells, α^2^ for the young cells was lower than that of the middle‐age and elderly cells. This indicates that despite R_b_ being high for the younger cells, the amount of adherence to the electrode was not as high. This could perhaps be explained by overcrowded younger cells, preventing tight adherence to the electrode surface.

Fluctuations in the collected impedance data were observed throughout this study (supplemental online Fig. 9). Movement of cells on the electrode can cause fluctuations in impedance readings 13, 14. In the current study, throughout the confluent stabilization phase, a significant amount of noise was associated with the resistance portion of the impedance data. During the impedance drop phase, there was an observed decrease in these fluctuations in the readings. To quantify the fluctuations in the data, we used signal entropy, which measures the amount of information contained within the signal 26. The higher the unpredictability of the signal data points, the higher its entropy value. It has been previously shown that mesenchymal stem cell motion is transiently upregulated in the early stages of osteogenic differentiation and decreased in late‐stage osteogenic differentiation 27. This is in agreement with the fluctuations captured in our impedance data and supports our conclusion that the impedance drop corresponds to final differentiation of the hASC. Furthermore, significant differences were observed between the entropy values of the superlots immediately upon osteogenic induction. Elucidation of these differences could be further developed as an early indicator for measuring donor‐specific hASC vitality and viability for tissue engineering procedures. Future studies should examine the micromotion of the hASC by using single electrodes and capturing changes in resistance data within 1‐Hz intervals and measured in periodic 1‐hour bursts to improve understanding of the dynamics of hASC motility both during and after differentiation.

This study used superlots instead of individual cell populations to determine the overall effects of age on the ability of hASCs to proliferate and differentiate down the osteogenic lineage. Superlots allow for an “average” effect of a variable (e.g., age) to be studied while reducing the noise introduced by individual variability unrelated to age 2. However, for this method to be developed into a technology for screening individual cell populations, hASCs from individual donors must be tested. Determining how individual hASC populations compare with the pooled results presented here should be studied in future investigations.

## Conclusion

This is the first study to use real‐time, noninvasive ECIS to elucidate and screen hASC proliferation and osteogenic variability between sex‐matched, age‐grouped donors. Importantly, different dielectric properties were observed among hASCs of different age groups. This suggests that ECIS may be used to screen hASCs isolated from different donors for osteogenic differentiation potential, providing a more thorough understanding of the quality of an hASC population than can be achieved by evaluating the population at a single time point. This technology could be incorporated into future bioreactors to track hASC through proliferation and differentiation to assist in quality control during stem cell manufacturing.

Through real‐time monitoring of the differentiation of different age group hASC superlots, we have found that hASCs from older donors differentiate down the osteogenic lineage in a shorter time frame than younger superlots, offering new insight into the known variability between hASC lines. The impedance drop phase preceded the first observed matrix deposition 48–96 hours after maximum impedance in all hASC superlots, suggesting that ECIS can detect cell morphology changes that correspond to late‐stage osteogenic differentiation. In addition, differences in micromotion could be discerned both between hASC superlots and between early‐ and late‐ stage differentiation, offering yet another method to evaluate and track hASC populations. The findings presented herein could be critical in developing patient‐specific bone tissue engineering and regenerative medicine therapies and better translating hASC therapies to clinical applications.

## Author Contributions

R.C.N.: conception and design, financial support, collection and/or assembly of data, data analysis and interpretation, manuscript writing, final approval of manuscript; J.Z.and M.W.F.: collection and/or assembly of data, data analysis and interpretation, final approval of manuscript; E.H.G.: data analysis and interpretation, manuscript writing, final approval of manuscript; B.S.: conception and design, financial support, data analysis and interpretation, manuscript writing, final approval of manuscript; E.G.L.: conception and design, financial support, manuscript writing, final approval of manuscript.

## Disclosure of Potential Conflicts of Interest

The authors indicated no potential conflicts of interest.

## Supporting information

Supporting Information
