# Supplementary material for: Electrical Cell‐Substrate Impedance Spectroscopy Can Monitor Age‐Grouped Human Adipose Stem Cell Variability During Osteogenic Differentiation
Source: Stem Cells Transl Med. 2016 Sep 7;6(2):502–11. doi: 10.5966/sctm.2015-0404 (PMC5442814; doi:10.5966/sctm.2015-0404)
Supplement: Supplementary file 1 — Supporting Information [file SCT3-6-502-s001.pdf]

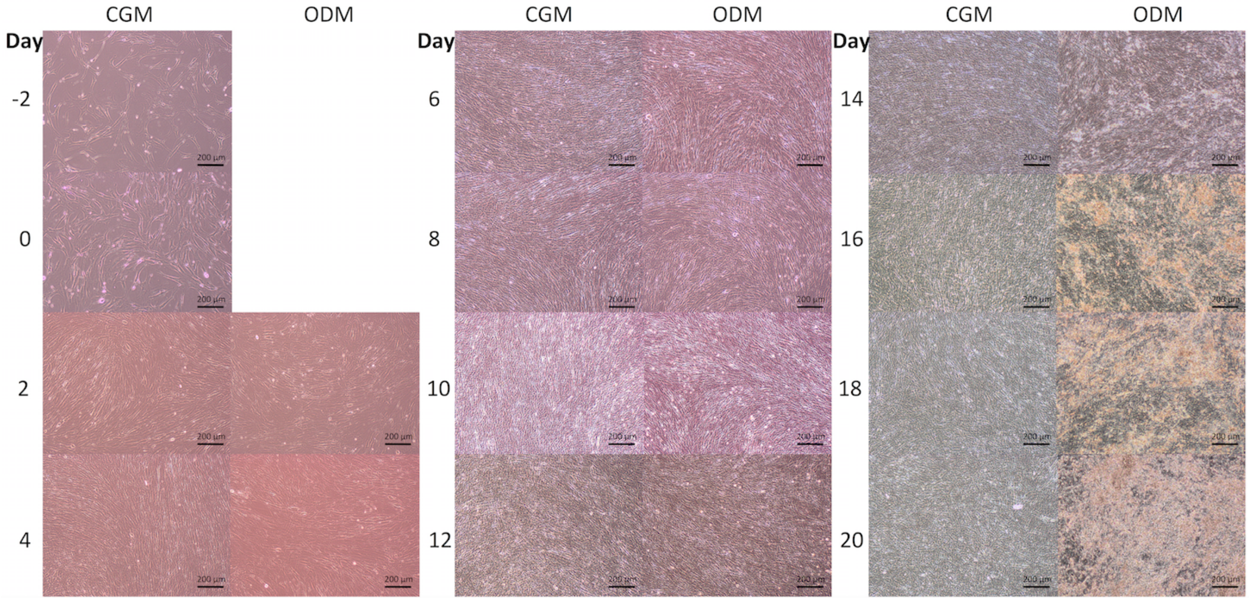

Supplementary Figure 1 Light microscope time course of young hASC cultured in both CGM and ODM. Matrix deposition was first observed at day 14 culture in ODM (scale bars = 200μm).

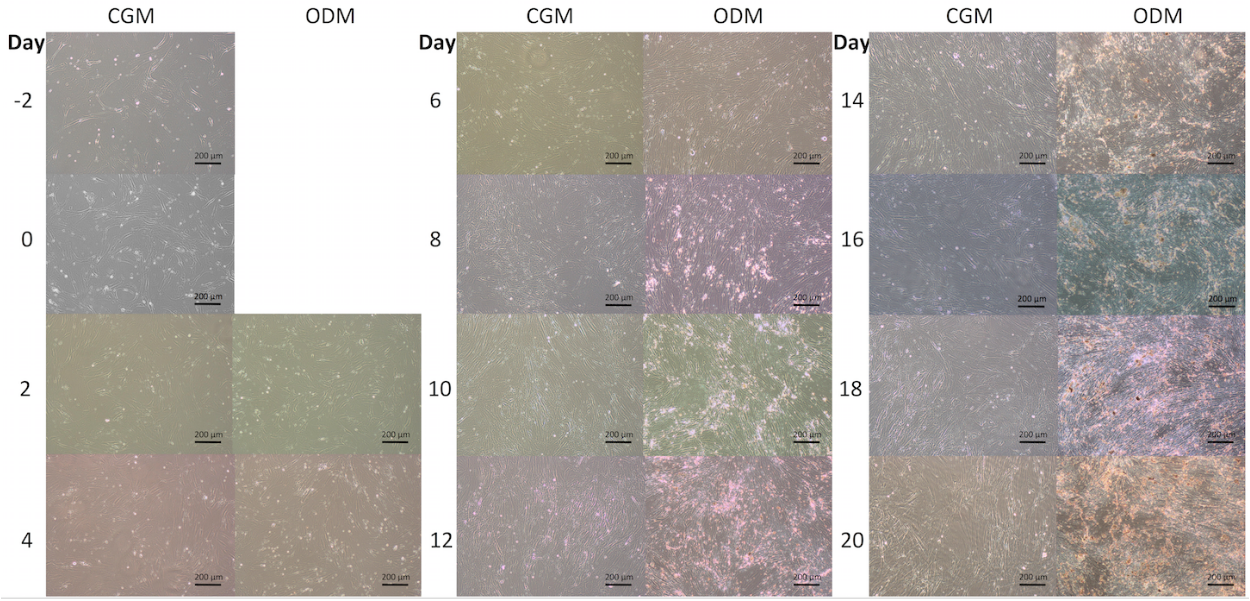

Supplementary Figure 2 Light microscope time course of middle-aged hASC cultured in both CGM and ODM. Matrix deposition was first observed at day 8 culture in ODM (scale bars = 200μm).

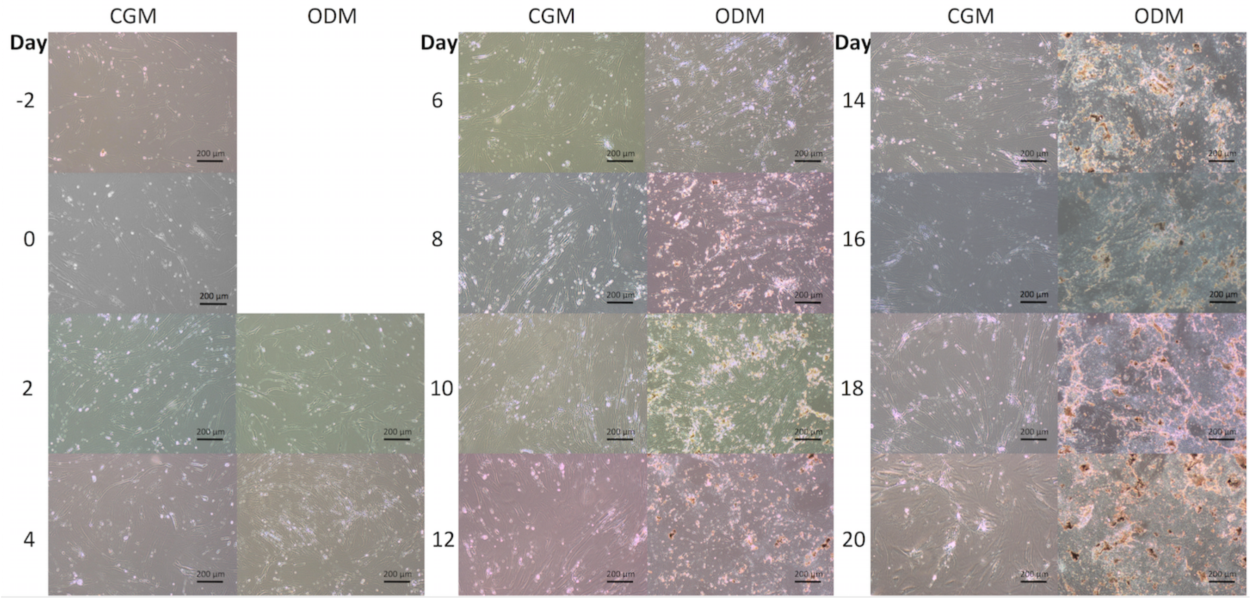

Supplementary Figure 3 Light microscope time course of elderly hASC cultured in both CGM and ODM. Matrix deposition was first observed at day 4 culture in ODM (scale bars = 200μm).

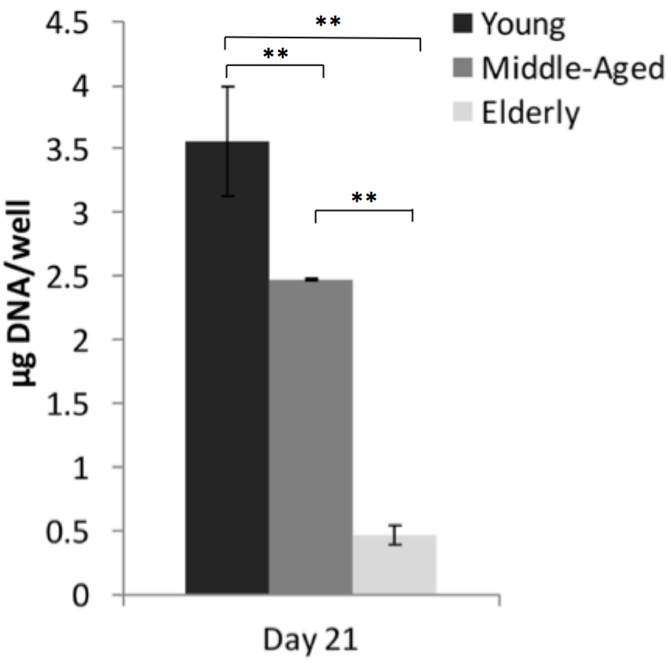

Supplementary Figure 4 Total DNA per well after 21 days of culture in ODM. DNA was quantified via Hoescht 33258 assay (Life Technologies, Carlsbad, CA). Significantly more DNA was observed in the younger superlot than middle-aged and elderly superlots. Results are in accordance with Alamar Blue data, which shows greatest percent reduction (ie. highest metabolic activity) in young superlot. The elderly superlot yielded low DNA, consistent with low percent reduction in Alamar Blue data (** = $p<0.01$).

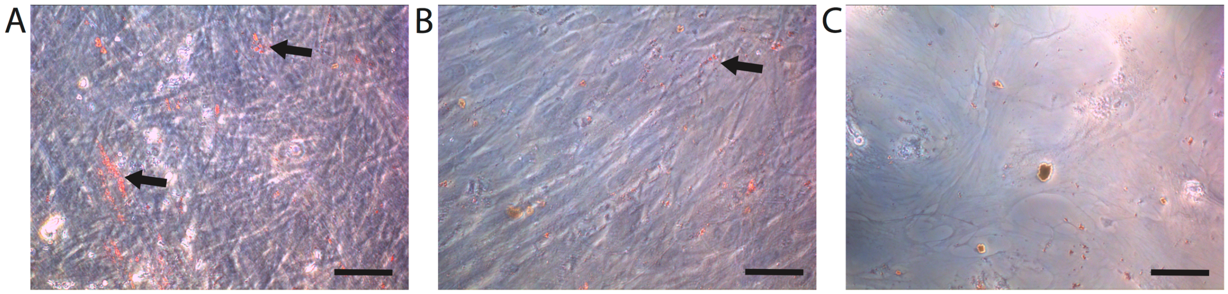

Supplementary Figure 5 Oil Red O staining for lipid accumulation after 21 days of osteogenic differentiation in **A.** young **B.** middle-aged and **C.** elderly superlots. There was little lipid accumulation in any superlot. However, more lipid accumulation was observed in the young superlot, followed by the middle-aged superlot. The elderly superlot did not exhibit any significant lipid accumulation (scale bars= 50μm).

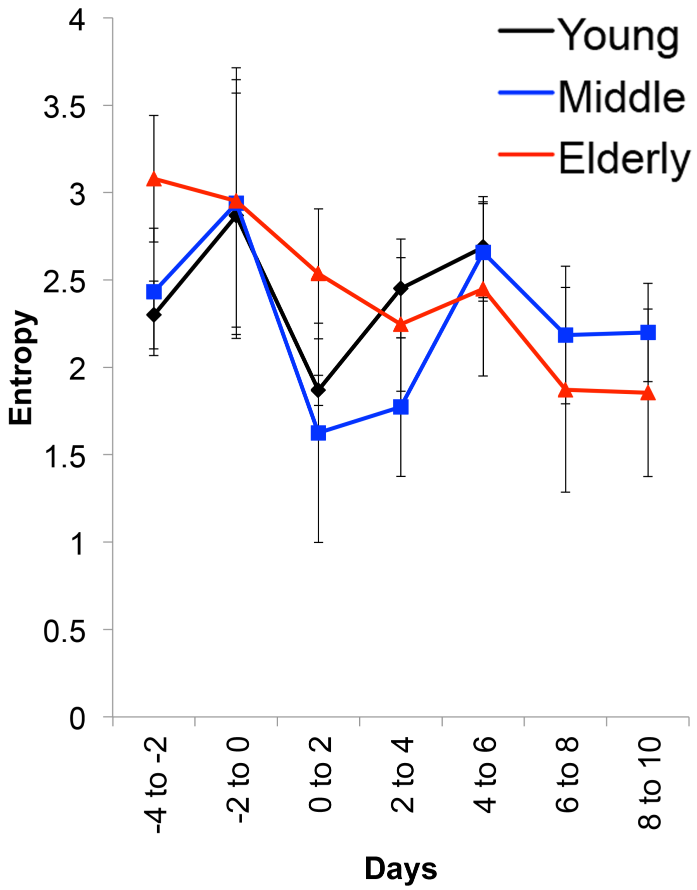

Supplementary Figure 6 No significant difference in signal entropy was observed between superlots cultured in CGM for the duration of the study.

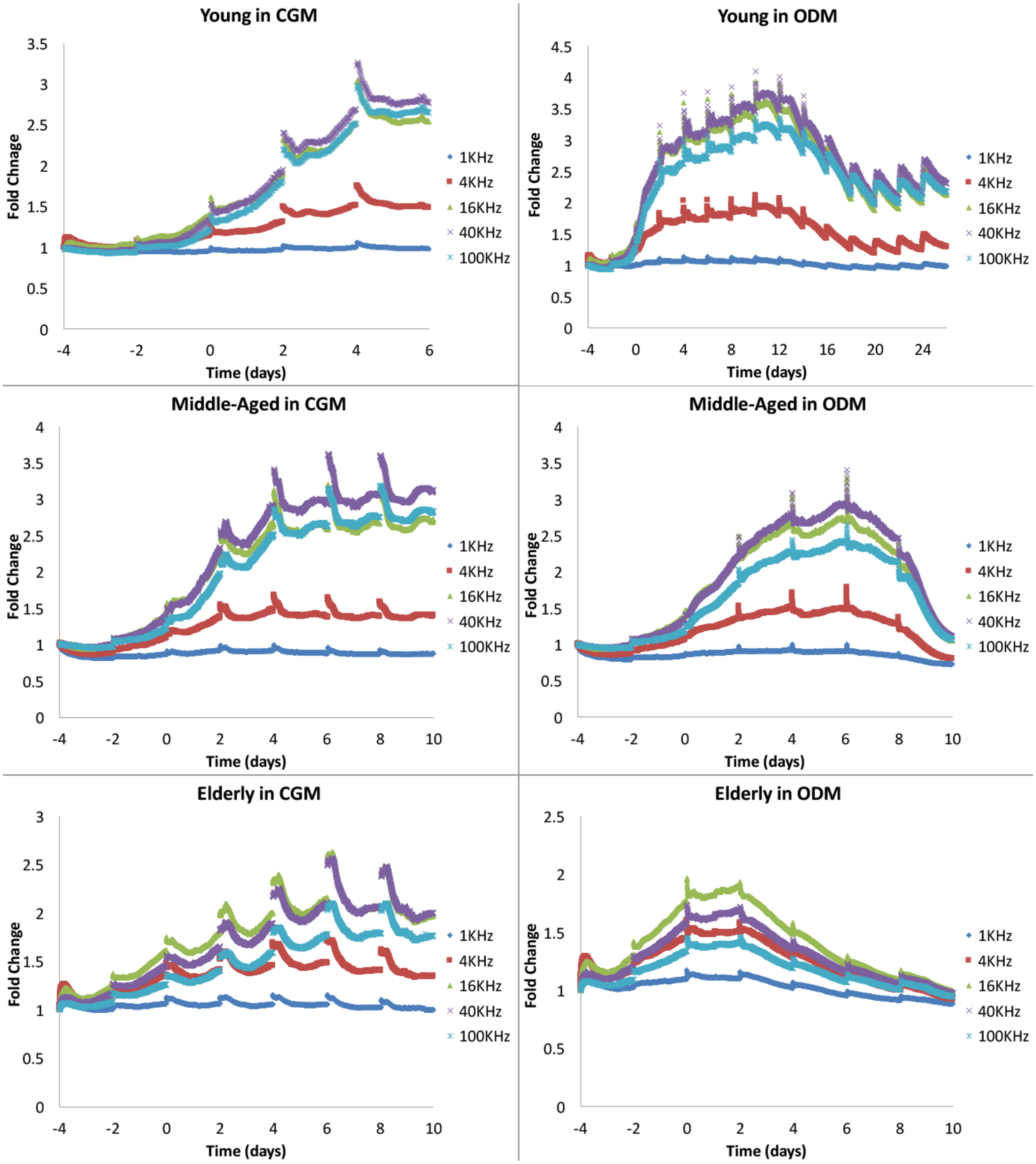

Supplementary Figure 7 A comparison of impedance data at different frequencies shows that similar trends are observed at both high and low frequencies. Data is normalized to starting impedance value of each data set.

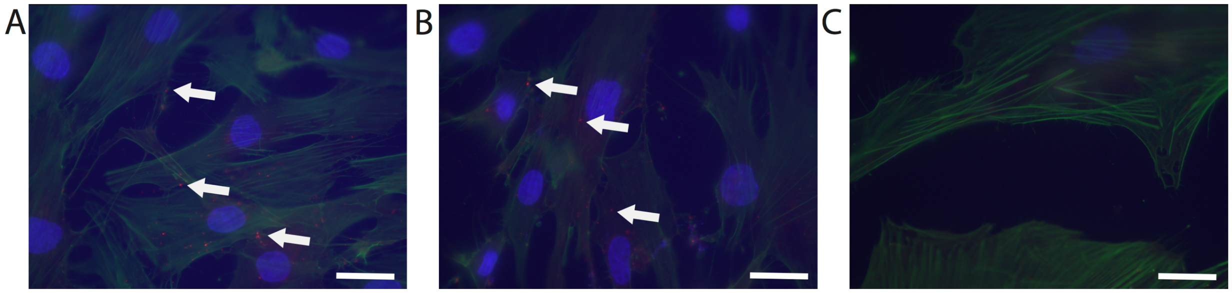

Supplementary Figure 8 Immunostaining of **A.** young **B.** middle-age and **C.** elderly superlots at time of induction (day 0). Stains used were DAPI (blue) (Invitrogen, Carlsbad, CA), phalloidin (green) (Invitrogen, Carlsbad, CA), and Cnx43 (red, arrows) (Abcam, Cambridge, United Kingdom). Cnx43 staining was observed in both young and middle-aged superlots. The elderly superlot did not show Cnx43 staining (scale bars= 50μm).

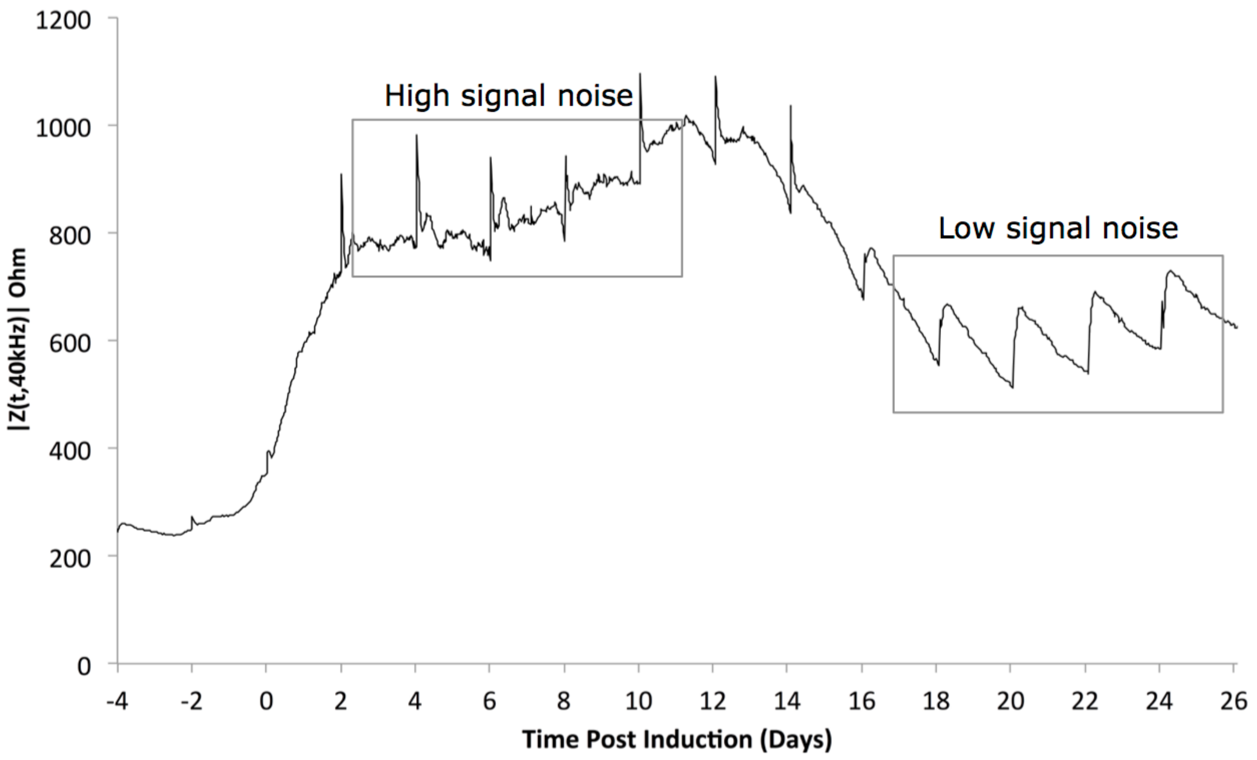

Supplementary Figure 9 A representative young superlot impedance data set. Higher signal noise was observed in the data before the impedance drop than after the impedance drop. The signal noise was quantified via signal entropy.
